# Drug Utilization Pattern of Antihypertensive Agents in a Tertiary Care Hospital

**DOI:** 10.7759/cureus.110328

**Published:** 2026-06-05

**Authors:** Leesha L Chawla, Vandana M Thorat, Kartiki P Patil

**Affiliations:** 1 Department of Pharmacology, Krishna Institute of Medical Sciences, Krishna Vishwa Vidyapeeth (Deemed to be University), Karad, IND

**Keywords:** antihypertensive agents, drug utilization, hypertension, prescribing patterns, who prescribing indicators

## Abstract

Introduction

Hypertension constitutes a significant global health challenge and is a major contributor to cardiovascular morbidity and death, particularly in economically developing nations. Although effective antihypertensive medications are widely accessible, satisfactory blood pressure regulation is often not achieved due to suboptimal prescribing practices. Periodic evaluation of how medications are prescribed and consumed in clinical practice is therefore necessary to bridge existing gaps and guide improvements in patient care. The primary objective of this study was to evaluate the drug utilization pattern of antihypertensive medications among hypertensive patients attending the outpatient department of a tertiary care hospital. The first secondary objective was to assess antihypertensive prescribing patterns in relation to World Health Organization (WHO) core prescribing indicators. The secondary objective was to examine adherence to the National List of Essential Medicines (NLEM) 2022 of India.

Methods

A cross-sectional observational study was conducted among 99 patients diagnosed with hypertension visiting the outpatient clinic of a tertiary care hospital over a period of 18 months, from March 2024 to August 2025. Patient information including sociodemographic characteristics, total number of medications prescribed, therapeutic drug categories and individual agents, dosing parameters, duration of antihypertensive treatment, and utilization of fixed-dose combinations (FDCs) were systematically documented. Medication use was assessed utilizing the standard WHO core prescribing indicators, and evaluation of medications sourced from the essential drug list was done using the NLEM 2022 of India.

Results

A total of 58 (58.6%) patients belonged to the ≥60-year age group, and 52 (52.5%) were males. Monotherapy was the most commonly prescribed treatment, accounting for 39 (39.4%) patients, followed by FDCs in 38 (38.4%) patients. Calcium channel blockers (CCBs) emerged as the most widely utilized drug class, prescribed in 65 (65.6%) patients. Among monotherapy recipients, CCBs and angiotensin receptor blockers (ARBs) were the predominantly used agents, accounting for 22 (22.2%) and 14 (14.1%) patients, respectively. Among free drug combinations, CCBs with ARBs were the most common, prescribed in 10 (10.1%) patients. Among FDCs, CCBs with ARBs were the most commonly prescribed in 11 (11.1%) patients, followed by CCBs with beta-blockers in seven (7%) and ARBs with diuretics in six (6.1%) patients. The mean number of drugs per prescription was 1.4±0.6, and 79 (58.9%) medications were drawn from the NLEM.

Conclusion

Antihypertensive prescribing patterns were broadly consistent with guideline-preferred first-line drug classes, with CCBs and ARBs as the prominent agents. A substantial proportion of medications were drawn from the NLEM, though the proportion prescribed outside the NLEM highlights the need for ongoing prescription monitoring. Periodic evaluation of antihypertensive prescribing trends in tertiary care hospital settings is recommended to inform quality improvement initiatives in hypertension management.

## Introduction

Hypertension represents one of the most pressing public health challenges worldwide and is among the foremost preventable contributors to cardiovascular disease-related illness and mortality. It is estimated that approximately 1.28 billion adults aged 30-79 years are affected by hypertension globally, with the majority residing in low- and middle-income countries [1]. In India, the prevalence and impact of hypertension have been rising consistently, largely driven by rapid urbanization, increasingly sedentary habits, changes in dietary patterns, and an aging population [2,3]. Despite effective antihypertensive therapies, satisfactory blood pressure management remains often not achieved, thereby elevating the risk of cerebrovascular events, acute coronary syndromes, cardiac failure, and renal insufficiency [4].

Effective pharmacological intervention is essential for achieving well-controlled blood pressure levels and minimizing related complications. A variety of antihypertensive drug classes, such as diuretics, calcium channel blockers (CCBs), angiotensin-converting enzyme inhibitors (ACEIs), angiotensin receptor blockers (ARBs), and beta-blockers, are commonly prescribed as single agents or in multi-drug regimens [5]. Nevertheless, issues such as irrational prescribing, excessive polypharmacy, and unsuitable drug combinations can adversely affect therapeutic outcomes and increase healthcare expenditures [6]. Hence, analyzing drug utilization patterns is critical to foster evidence-based prescribing behavior and improve patient care.

As part of its global health mandate, the World Health Organization (WHO) has established core prescribing metrics designed to evaluate rational drug use, including metrics such as the mean count of medications per prescription, the proportion of medicines sourced from essential medicine lists, and the prescription of fixed-dose combinations (FDCs) [7]. In the Indian context, the National List of Essential Medicines (NLEM) of India serves as an important framework to encourage judicious and evidence-based medication use [8]. Investigations into prescribing patterns yield valuable information regarding prevailing practices and highlight opportunities for clinical improvement [9]. Furthermore, the WHO defines drug utilization research as the study of the procurement, supply chain, prescription patterns, and consumption of medicines across communities, with particular focus on their medical, social, and health-economic implications [10].

Drug utilization research is a valuable tool for evaluating medication use patterns, improving prescription quality, and reducing disease burden and associated costs. Such data are also essential for examining annual expenditure on drug procurement, assessing the adequacy of drug supply to patients, analyzing medication pricing, and evaluating overall patterns of drug consumption and utilization [11].

Considering the growing burden of hypertension and the inherent complexities involved in its management, a comprehensive evaluation of prescribing trends in real-world scenarios is warranted. The primary objective of this study was to evaluate the drug utilization pattern of antihypertensive medications among hypertensive patients attending the outpatient department of a tertiary care hospital. The first secondary objective was to assess antihypertensive prescribing patterns in relation to WHO core prescribing indicators. The secondary objective was to examine adherence to the NLEM 2022 of India. We hypothesized that antihypertensive prescribing practices in this tertiary care setting would be broadly consistent with guideline-recommended antihypertensive therapy and that a substantial proportion of prescribed medications would be sourced from the NLEM.

## Materials and methods

Study design and population

The present investigation was designed as a cross-sectional observational study conducted among 99 individuals with a confirmed diagnosis of hypertension who were on antihypertensive treatment for over one month, presenting to the outpatient clinic of medicine in Krishna Charitable Hospital and Medical Research Centre, Krishna Institute of Medical Sciences (KIMS), Krishna Vishwa Vidyapeeth (Deemed to be University), Karad, Maharashtra, India. Data collection was conducted over 18 months, from March 2024 to August 2025. The study was conducted after obtaining approval from the Institutional Ethics Committee of Krishna Vishwa Vidyapeeth (Deemed to be University), Karad (approval number: KVV/IEC/05/2024; protocol number: 349/2023-2024). Patients were recruited using consecutive sampling, whereby all eligible patients attending the outpatient department during the study period who met the inclusion criteria were enrolled until the required sample size was achieved. For each enrolled patient, data were recorded from the prescription issued at the time of the outpatient visit during the study period. Only one prescription per patient encounter was included in the analysis to avoid duplication. Repeat visits of the same patient during the study period were excluded from the analysis to prevent duplicate data entry.

Inclusion Criteria

Patients aged 18 years and above irrespective of their gender who had been diagnosed with hypertension and were receiving antihypertensive treatment for over one month were enrolled after taking written informed consent.

Exclusion Criteria

Patients with cognitive impairment or psychiatric disorders, pregnant or lactating women, and patients unwilling to participate were excluded from the study. 

Sample size

Sample size was independently calculated for each drug class using their respective prevalence values reported by Adejumo et al. [12]. Among all drug classes, the prevalence of CCB use (54.9%) yielded the highest sample size estimate and was therefore selected as the reference value for the final sample size calculation, ensuring adequate representation and statistical power for the analysis of all antihypertensive drug classes. Sample size estimation was performed using the following formula: $\mathrm{N}=\mathrm{Z}^{2}\mathrm{pq}/\mathrm{L}^{2}$. Here, N represents the required sample size, Z denotes the standard normal deviate at a 95% confidence interval (2), p represents the prevalence of antihypertensive agents (54.9% for CCBs) based on a study by Adejumo et al. [12] ($\mathrm{q}=100-\mathrm{p}=45.1\%$), and L denotes the precision set at 10%. Substituting the above values in the formula, $\mathrm{n}=(2)^{2}\times0.549\times0.451/(0.10)^{2}=4\times0.2476/0.01=99.03$, which was rounded up to 99. The rounding from 99.03 to 99 was considered statistically acceptable given the negligible difference, and no additional adjustment for non-response or loss was required as data were collected prospectively from outpatient prescriptions with complete records verified at the time of collection, yielding a final sample of 99 patients.

Data collection and assessment 

Data collection was carried out on all scheduled outpatient clinic days during the study period. Patient information was gathered through a predesigned case record form comprising the following domains: patient demographics (age, sex, weight, height, body mass index (BMI)), clinical parameters (blood pressure in mmHg, stage of hypertension), medical history (history of allergy, past major illness, family history, personal history), comorbid illnesses, and prescription details (total number of drugs prescribed, individual drug names, doses, routes and frequency of administration, and use of FDCs). Prescription sheets were systematically reviewed, and relevant variables were recorded. Information regarding blood pressure stage, BMI, and comorbidities was obtained from patient case records and outpatient prescription documentation recorded during the clinic visit. Hypertension staging was classified according to the guidelines of the Eighth Joint National Committee (JNC8), wherein stage 1 hypertension was defined as systolic blood pressure of 140-159 mmHg or diastolic blood pressure of 90-99 mmHg and stage 2 hypertension was defined as systolic blood pressure of ≥160 mmHg or diastolic blood pressure of ≥100 mmHg. Blood pressure values were recorded from medical case records at the time of the outpatient visit. Comorbid conditions were identified through chart-based review of medical records, including documented physician diagnoses, prescription histories, and available laboratory reports. Patients with comorbid conditions and those receiving concomitant medications alongside antihypertensive therapy were included in the study, as excluding such patients would not reflect real-world prescribing practices. However, only antihypertensive medications were evaluated for the purpose of drug utilization analysis. Monotherapy was defined as the use of a single antihypertensive agent for blood pressure control. Free-drug combination therapy referred to the concurrent use of two or more separately prescribed antihypertensive drugs, whereas FDC therapy referred to the use of two or more antihypertensive agents combined within a single formulation. Prescribing behavior and drug use were analyzed using the standard WHO prescribing indicators. As this study focused on antihypertensive drug utilization, the WHO core prescribing indicators were adapted to evaluate antihypertensive medications rather than all prescribed drugs. The average number of drugs per prescription was calculated by dividing the total number of drugs prescribed by the total number of prescriptions analyzed. Evaluation of medications sourced from the essential drug list was done using the NLEM 2022 of India. Prescribed medications were cross-referenced against the NLEM 2022 of India to assess adherence to essential medicine prescribing.

Statistical analysis

All data were recorded in Microsoft Excel 2021 (Microsoft Corporation, Redmond, Washington, United States), and statistical analysis was performed using IBM SPSS Statistics for Windows, Version 27.0 (IBM Corp., Armonk, New York, United States). Data was analyzed using descriptive statistics. Categorical variables were represented in the form of percentages and frequencies, while continuous variables were summarized using mean and standard deviation where applicable.

## Results

Sociodemographic characteristics

Table 1 describes the sociodemographic profile of the patients. The largest proportion of participants, i.e., 58 (58.6%), fell within the age category of ≥60 years, with 52 (52.5%) being male. With respect to educational status, the majority of patients, i.e., 68 (68.7%), were below graduate level.

**Table 1:** Sociodemographic characteristics

Variables	N (%)
Age (years)	
18-39	4 (4)
40-59	37 (37.4)
≥60	58 (58.6)
Gender	
Male	52 (52.5)
Female	47 (47.5)
Education	
Illiterate	16 (16.2)
Below graduate	68 (68.7)
Graduate and above	15 (15.2)

Clinical and disease profile

Table 2 describes the clinical and disease profile of the patients enrolled in the study. With regard to BMI, 54 (54.5%) subjects had a normal BMI, 29 (29.3%) were overweight, 12 (12.1%) were underweight, and four (4%) were obese. Stage 1 hypertension was more prevalent, observed in 60 (60.6%) patients, compared to stage 2 hypertension, which was recorded in 39 (39.4%) patients. Treatment duration was <5 years in 52 (52.5%) patients, 5-10 years in 31 (31.3%) patients, and >10 years in 16 (16.2%) patients. A total of 48 (48.5%) patients presented without any comorbidities. Among those with comorbid conditions, ischemic heart disease was the most common, recorded in 24 (24.2%) patients, followed by diabetes mellitus in 20 (20.2%) patients, while chronic kidney disease was recorded in two (2%) patients, and other conditions were noted in five (5.1%) patients.

**Table 2:** Clinical and disease profile BMI: body mass index; DM: diabetes mellitus; CKD: chronic kidney disease; IHD: ischemic heart disease

Variables	N (%)
BMI (kg/m²)	
Underweight (<18.5)	12 (12.1)
Normal (18.5-24.99)	54 (54.5)
Overweight (25-29.99)	29 (29.3)
Obese (≥30)	4 (4)
Stage of hypertension	
Stage 1	60 (60.6)
Stage 2	39 (39.4)
Duration of treatment (years)	
<5	52 (52.5)
5-10	31 (31.3)
>10	16 (16.2)
Comorbidities	
None	48 (48.5)
DM	20 (20.2)
CKD	2 (2)
IHD	24 (24.2)
Others	5 (5.1)

Therapy pattern

**Figure 1:**
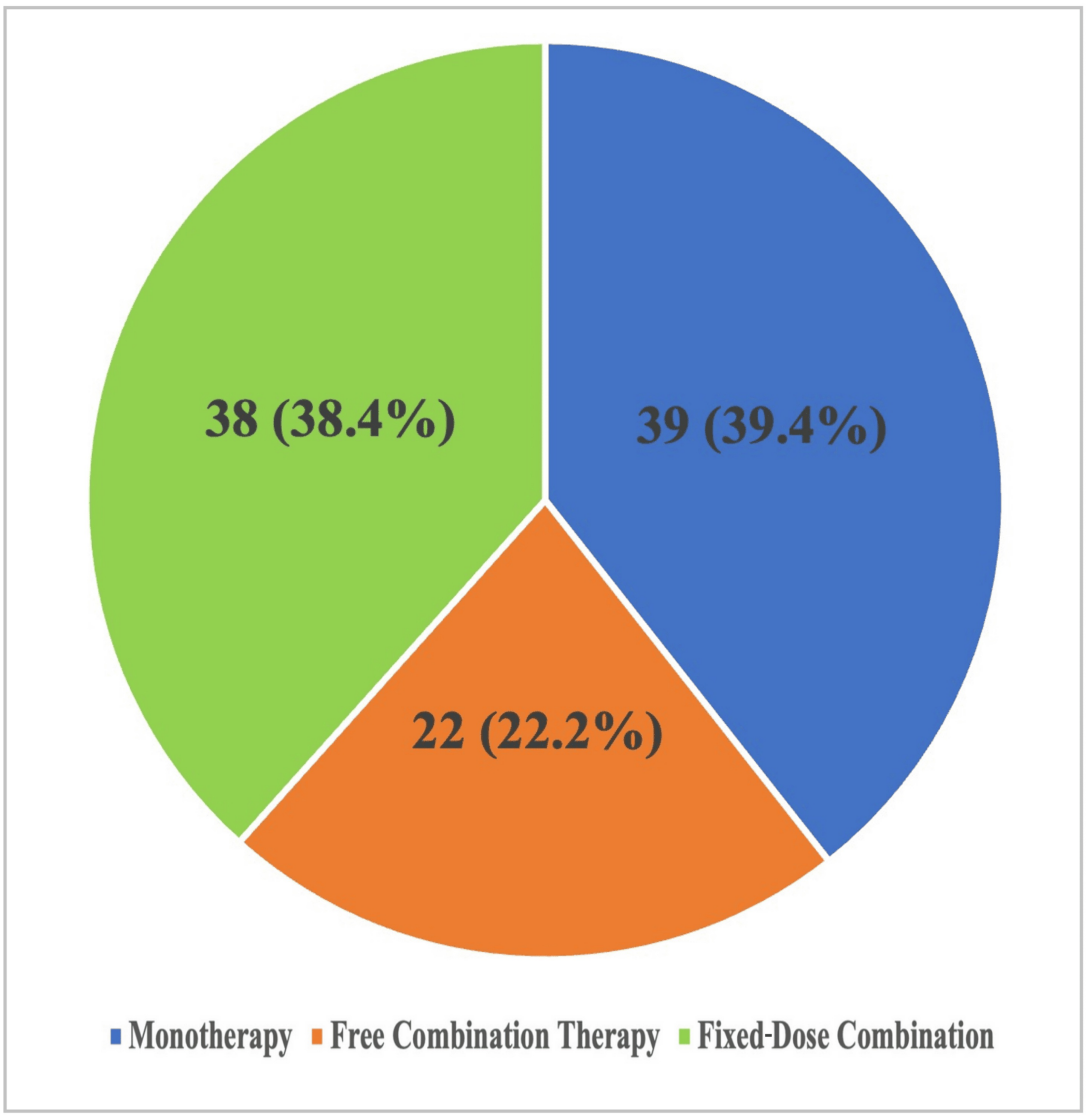
Antihypertensive therapy pattern Pie chart depicting the distribution of antihypertensive therapy patterns among the study participants. Data are presented as number (percentage).

Monotherapy was the most common regimen, recorded in 39 (39.4%) patients, followed by FDCs in 38 (38.4%) patients and free combinations in 22 (22.2%) patients, as illustrated in Figure 1.

Major antihypertensive agents 

**Figure 2:**
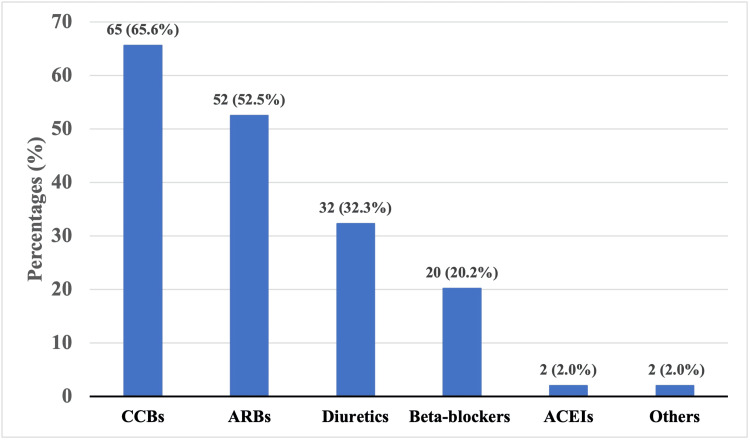
Distribution of major antihypertensive agents Bar graph illustrating the distribution of major antihypertensive agents. Data are presented as number (percentage). CCBs: calcium channel blockers; ARBs: angiotensin receptor blockers; ACEIs: angiotensin-converting enzyme inhibitors

Figure 2 depicts the distribution pattern of major antihypertensive agents. CCBs were the most frequently prescribed drug class, recorded in 65 (65.6%) patients, followed by ARBs in 52 (52.5%) patients, diuretics in 32 (32.3%) patients, and beta-blockers in 20 (20.2%) patients. ACEIs and other agents were rarely prescribed, with each accounting for two (2%) patients.

Drug utilization pattern

The results regarding antihypertensive prescribing among patients were represented in Table 3. CCBs and ARBs were the most frequently utilized drug class agents among monotherapy recipients, accounting for 22 (22.2%) and 14 (14.1%) patients, respectively. Combination therapy was widely practiced, reflecting the need for multi-drug regimens to achieve optimal blood pressure control. Among free drug combinations, CCBs with ARBs were the most common, recorded in 10 (10.1%) patients, highlighting their complementary mechanisms. Among FDCs, CCBs with ARBs were the most commonly prescribed, observed in 11 (11.1%) patients, followed by CCBs with beta-blockers in seven (7%) patients and ARBs with diuretics in six (6.1%) patients, indicating an emphasis on improving adherence and therapeutic efficacy. Overall, the findings underscore a strong inclination toward combination therapy, especially CCB- and ARB-based regimens, in contemporary clinical practice.

**Table 3:** Antihypertensive drug utilization pattern CCBs: calcium channel blockers; ACEIs: angiotensin-converting enzyme inhibitors; ARBs: angiotensin receptor blockers; FDCs: fixed-dose combinations

Antihypertensive drug class	N (%)
Monotherapy	
CCBs	22 (22.2)
ACEIs	1 (1)
ARBs	14 (14.1)
Beta-blockers	2 (2)
Free drug combination therapy	
CCBs+ARBs	10 (10.1)
CCBs+beta-blockers	3 (3)
Diuretics+CCBs	2 (2)
ARBs+beta-blockers	1 (1)
CCBs+alpha-blockers	1 (1)
Diuretics+ARBs	1 (1)
Beta-blockers+central sympatholytics	1 (1)
Diuretics+CCBs+ARBs	1 (1)
CCBs+ARBs+beta-blockers	1 (1)
Loop diuretics+potassium-sparing diuretics+ARBs+beta-blockers	1 (1)
FDC therapy (FDC alone)	
Loop diuretics+potassium-sparing diuretics	5 (5)
CCBs+ARBs	11 (11.1)
CCBs+beta-blockers	7 (7)
CCBs+diuretics	2 (2)
ARBs+diuretics	6 (6.1)
ARBs+beta-blockers	1 (1)
FDC therapy (FDC with other drugs)	
(Loop diuretics+potassium-sparing diuretics)+CCBs	1 (1)
(Loop diuretics+potassium-sparing diuretics)+beta-blockers	1 (1)
(Loop diuretics+potassium-sparing diuretics)+CCBs+ARBs	1 (1)
(CCBs+ARBs)+beta-blockers	2 (2)
(ARBs+diuretics)+ACEIs	1 (1)
(ARBs+diuretics)+CCBs	1 (1)

WHO core prescribing indicators

Table 4 describes the standard WHO prescribing indicators. An aggregate of 99 prescriptions was reviewed and analyzed, comprising 134 antihypertensive drugs, with a mean of 1.4±0.6 drugs per prescription. Seventy-nine (58.9%) medications were drawn from the NLEM, and 38 (38.4%) patients were prescribed with FDC.

**Table 4:** WHO core prescribing indicators WHO: World Health Organization; NLEM: National List of Essential Medicines; FDC: fixed-dose combination

WHO prescribing indicators	Values
Total prescriptions (N)	99
Total drugs prescribed (N)	134
Average drugs per prescription (mean±SD)	1.4±0.6
Drugs from NLEM (%)	58.9
Patients on FDC (%)	38.4

## Discussion

There is limited evidence from resource-constrained settings examining drug utilization patterns in hypertension management. In view of the increasing prevalence of hypertension and its related complications, evaluating real-world prescribing trends becomes essential [13]. Therefore, this study was conceived to assess antihypertensive prescribing trends among patients attending a tertiary hospital setting.

The demographic profile of enrolled patients revealed that a majority, 58 (58.6%) patients, were aged ≥60 years, followed by 37 (37.4%) patients in the 40-59-year category, suggesting a greater prevalence of hypertension in the elderly. This finding corroborates published evidence identifying advancing age as a key risk factor due to vascular remodeling and increased arterial stiffness [14]. A slight male predominance was noted in our cohort, with 52 (52.5%) being male, consistent with prior epidemiological data demonstrating greater hypertension rates documented in men in middle-aged and older groups [15]. With respect to educational background, the majority of subjects, 68 (68.7%), had education below the graduate level, suggesting that limited educational achievement may influence awareness and medication-taking behavior, as reported in previous literature [16].

Assessment of clinical features revealed that the majority, 54 (54.5%), of enrolled patients had a normal BMI, while 29 (29.3%) were overweight, underscoring the contribution of lifestyle-related factors to hypertension. Similar findings have been reported in studies highlighting a well-established relationship between elevated BMI and increased hypertension risk [17,18]. Stage 1 hypertension was more prevalent, recorded in 60 (60.6%) patients, than stage 2 which was observed in 39 (39.4%) patients, suggesting that a considerable number of patients had been identified at an earlier stage, offering scope for timely intervention and prophylaxis against complications [19]. Additionally, over half of the participants, 52 (52.5%), had been receiving antihypertensive therapy for a duration of <5 years, reflecting relatively recent diagnosis. A total of 48 (48.5%) patients had no comorbid conditions, whereas ischemic heart disease and diabetes mellitus were the most frequently observed associated illnesses, consistent with the clustering of cardiovascular risk factors [20].

Evaluation of prescribing trends revealed that monotherapy, recorded in 39 (39.4%) patients, and FDCs, observed in 38 (38.4%) patients, were employed with comparable frequency. This pattern aligns with guideline-based recommendations, which support multi-drug regimens in patients with inadequately controlled blood pressure [20]. Among drug classes, CCBs constituted the most prescribed category, recorded in 65 (65.6%) patients, followed by ARBs, observed in 52 (52.5%) patients. The high utilization of CCBs and ARBs observed in this study is clinically appropriate given the predominantly elderly study population, as current hypertension management guidelines recommend CCBs as preferred first-line agents in elderly patients, while ARBs are particularly indicated in those with comorbid conditions such as diabetes and chronic kidney disease, owing to their favorable efficacy and tolerability profiles. These findings are in concordance with earlier reports citing these classes as commonly utilized [21,22]. In the present study, diuretics, beta-blockers, and ACEIs were prescribed less frequently. This lower utilization frequency is consistent with contemporary hypertension management guidelines, which reserve beta-blockers and ACEIs primarily for individuals presenting with select comorbid diagnoses including coronary artery disease, cardiac failure, or intolerance to other first-line antihypertensive agents [23]. Similar patterns of selective prescribing have likewise been documented across several antihypertensive drug utilization studies [24,25]. The limited use of ACEIs, central sympatholytic agents, and alpha-blockers further indicates individualized prescribing practices tailored to patient-specific clinical profiles. Overall, the antihypertensive prescribing pattern identified in the present study was broadly in keeping with evidence-based hypertension management principles, demonstrating the rational selection of first-line medications along with the judicious use of adjunctive therapies.

Assessment of WHO core prescribing indicators provides an objective measure for evaluating the rationality of medication use in clinical practice. In this study, the observed prescription behavior for antihypertensive agents largely reflected rational pharmacotherapy practices. The mean count of antihypertensive agents per prescription was 1.4±0.6, indicating a relatively low degree of polypharmacy. Comparable results have been documented in other tertiary care drug utilization research carried out in India [26,27]. While 79 (58.9%) antihypertensive medications were drawn from the NLEM, the remaining 55 (41.1%) were prescribed from outside the essential medicines list. This may be attributed to several factors including prescriber preference for newer antihypertensive agents with favorable pharmacological profiles, availability of specific drug formulations within the hospital formulary, cost considerations, and patient-specific clinical requirements. A comparable level of adherence to essential medicine prescribing has also been observed by Sharma et al. [28]. FDCs were utilized among 38 (38.4%) patients. The judicious application of FDCs is widely recommended in hypertension management to minimize pill burden and improve patient adherence. Similar trends toward an increased rise in multi-drug combination use have been noted by Karki et al. [29]. As this investigation was carried out in an outpatient setting, no injectable antihypertensive agents were prescribed, which is consistent with rational prescribing practices reported by Shankpal et al. [27].

The clinical significance of this study lies in its integrated evaluation of prescribing practices within a real-world clinical setting. From a clinical standpoint, optimizing drug utilization can substantially reduce the incidence of hypertension-related complications. From a public health perspective, encouraging rational prescribing can improve clinical outcomes while simultaneously reducing unnecessary healthcare costs.

Certain limitations of the present investigation should be acknowledged. Being conducted at a single tertiary institution, findings may have limited applicability to the wider population, and formal recruitment data including refusal rates were not systematically recorded, limiting the formal assessment of selection bias. Although the sample size was adequate for descriptive analysis, it may not provide sufficient statistical power for inferential comparisons. Furthermore, confidence intervals were not reported for key proportions, which limits the precision of estimates. Larger multi-site investigations are needed to yield more robust insights into prescribing patterns. The cross-sectional design limits the ability to establish temporal relationships or causality between prescribing practices and clinical outcomes, and longitudinal studies would be more informative in assessing temporal trends and the direct impact of therapeutic interventions. Furthermore, clinical outcomes such as blood pressure control and medication adherence were not directly measured, limiting the ability to establish a direct association between the observed prescribing patterns and therapeutic outcomes. The WHO prescribing indicators were adapted to specifically focus on antihypertensive medications, which may limit comparability with studies using the full standard WHO indicator set. Furthermore, the percentage of antihypertensive medications prescribed by generic name was not assessed, and its absence limits the completeness of the prescribing pattern evaluation. Additionally, prescriber-level factors such as clinical experience, as well as hospital formulary constraints, were not assessed. The timing of initial hypertension diagnosis was not assessed; only the duration of antihypertensive therapy was recorded, which may not accurately reflect the actual duration of the disease. 

## Conclusions

The study cohort was predominantly composed of elderly individuals with a broadly balanced gender distribution and a moderate level of educational attainment. Most individuals had a normal BMI, although a notable proportion was overweight, underscoring the presence of weight-related cardiovascular risk factors within this population. About half of the patients exhibited no concomitant comorbidities, and among those with comorbid conditions, ischemic heart disease and diabetes mellitus were the predominant conditions.

The prescribing of antihypertensive therapy demonstrated a balanced utilization of monotherapy and FDC therapies, with overall trends broadly consistent with guideline-recommended first-line agents, particularly CCBs and ARBs. The relatively low mean antihypertensive drug count per prescription indicated a minimal tendency toward polypharmacy. A substantial proportion of medications were drawn from the NLEM of India, though the remaining proportion prescribed outside the NLEM highlights the need for ongoing prescription monitoring in similar settings. These findings should be interpreted within the context of a single-center cross-sectional study design, and extrapolation to broader populations should be made with caution. Periodic evaluation of antihypertensive prescribing trends in tertiary care hospital settings is recommended to inform quality improvement initiatives in hypertension management.
